# Evaluation of a synchronous training program on common primary care medications for community health workers in Karnataka, India

**DOI:** 10.1186/s12913-025-13754-x

**Published:** 2026-02-04

**Authors:** Ashwini Deshpande, Ananth Ram, Renuka Shanmugam, Kothandan Kumar, Muruga Munimada, Nayanjeet Chaudhury

**Affiliations:** 1 Department of Medicine, Stanford University School of Medicine, Stanford, CA 94305 USA; 2 Ramaiah International Center for Public Health Innovations, MS Ramaiah Nagar, MSRIT Post, Bangalore, 560097 India; 3 Jothydev’s Diabetes Research Center, JDC Junction, Mudavanmugal, Konkalam, Trivandrum, Kerala 695032 India; 4 Department of Community Medicine, Ramaiah Medical College, Bengaluru, India

**Keywords:** Capacity building, Chronic disease, Community health workers, Primary care medications

## Abstract

**Background:**

Community health workers (CHWs) are a sustainable solution for chronic disease care in rural India. Though CHWs often face medication-related questions from patients, they do not receive standardized training on primary care medications. In our study, we delivered a workshop on medication use, mechanism of action, appropriate dosage, and side effects to 147 CHWs in two districts of rural Karnataka and assessed its effectiveness in increasing the knowledge, self-efficacy, and confidence of participating CHWs.

**Methods:**

This quasi-experimental study evaluated a workshop that was collaboratively designed with stakeholders from primary health centers (PHCs)—including medical officers and community health workers (CHWs)—to enhance CHWs’ medication-related knowledge, confidence and self-efficacy. One workshop covered 64 CHWs in Ramanagara district, while four workshops together covered 83 CHWs in Madhugiri. Participants completed a pre-workshop and post-workshop assessment of medication-related knowledge, self-efficacy and confidence. The pre- and post-workshop median knowledge scores and proportion of CHWs with high self-efficacy and confidence were compared using Wilcoxon’s signed rank test and McNemar test, respectively.

**Results:**

Overall, 140 of the 147 (95.2%) participating CHWs completed the pre- and post-workshop assessments. More than 87% of CHWs reported medication-related responsibilities, but fewer than 33% had received information on medications in the past year. After attending the workshop, the median knowledge scores out of 20 increased from 11 to 16 (*p* < 0.01), and there was a statistically significant improvement in self-efficacy and confidence in completing medication-related responsibilities. Over 92% of CHWs found the training engaging, easy to understand, and relevant to their duties.

**Conclusions:**

This workshop was successful in providing CHWs with a baseline level of knowledge and confidence to manage medication regimens, particularly when CHWs are often confronted with medication-related tasks. This workshop provided the foundational knowledge for CHWs to better promote medication adherence and monitor for side effects.

**Supplementary Information:**

The online version contains supplementary material available at 10.1186/s12913-025-13754-x.

## Background

Cardiovascular disease is the leading cause of mortality and disability in India [1]. According to the Global Burden of Disease study, India’s age-standardized cardiovascular disease mortality rate is 272 per 100,000 population, exceeding the global average of 235 per 100,000 [2]. This higher-than-average mortality burden is further compounded by India’s rapid epidemiological transition from predominantly infectious diseases to noncommunicable diseases over a relatively short period of time [3].

Two of the largest risk factors for cardiovascular disease include diabetes and hypertension. Though approximately 20% of Indians in rural communities have been diagnosed with high blood pressure, only about 40% of those diagnosed are able to keep their blood pressure under control [4, 5]. Poor management of hypertension in rural communities could be due to a host of factors including geographical isolation from quality healthcare, poor health literacy, lack of trust in healthcare providers, and improper adherence to medication regimens [6]. This is also due to the fact that limited access to healthcare, transportation, and healthy food can create more vulnerable communities in rural areas [7]. There are critical primary healthcare workforce (e.g. physicians and nurses) shortages particularly in rural communities, highlighting the need for non-traditional healthcare providers to aid community members in chronic disease management [8, 9].

Specifically, community health workers (CHWs) can extend healthcare services to traditionally underserved rural communities by spearheading efforts to prevent, screen, diagnose, and manage chronic disease in these settings. Previous training programs for CHWs have been successful in increasing knowledge and procedural competency in care areas such as hypertension and chronic respiratory disease management [10]. Additionally, CHW services are cost-effective within the healthcare system [11]. CHWs have generally built strong interpersonal relationships with community members, leading to increased community trust in CHWs than in traditional healthcare providers [12, 13].

According to Ayushman Bharat Comprehensive Primary Health Care (CPHC) guidelines through Health and Wellness Centers (HWCs), the Community Health Officers (CHOs) who are nursing graduates and the Primary Health Care Officers (PHCOs) who are trained health workers are expected to dispense follow-up medication as prescribed by the Medical Officer at Primary Healthcare Centre (PHC) or Community Health Centre (CHC). They are also required to ensure medication compliance among patients in the community with chronic noncommunicable diseases [14]. Unfortunately, there are no guidelines to standardize training requirements specific to the medications that are available at the primary care level for CHWs [15]. CHWs may be tasked with medication management; however, due to variability in their training, CHWs may not feel prepared to manage complex medication regimens in chronic disease patients [10, 15]. Thus, we sought to create an optimal training model to expand CHWs’ knowledge of commonly used medications in the management of diabetes, hypertension, and heart disease medications in rural Indian settings so that CHWs are better prepared to counsel chronic disease patients.

In this paper, we have attempted to assess the effectiveness of synchronous, workshop-based training in increasing CHWs’ knowledge of medications, self-efficacy, and confidence in their work profiles in two districts of rural Karnataka, India.

## Methods

The study was conducted between March 2021 and July 2023 and was approved by the Institutional Review Board at M.S. Ramaiah Medical College under the protocol number *{MSRMC/EC/AP-09/02-2022}*. This is a quasi-experimental study evaluating the effectiveness of a medication-related training workshop for community health workers in rural Karnataka, India.

### Needs assessment

The research team conducted a preliminary needs analysis by interviewing the healthcare providers of urban and rural PHCs, CHWs that included PHCOs, CHOs, and health administrators at the sub-divisional and district levels about training gaps regarding medications available at HWCs and PHCs (Supplemental file 1). Later, the team listed primary care medications available at the facilities and reviewed the essential drug list at primary healthcare delivery levels at PHCs and HWCs.

### Design workshop and prototype development

Based on the findings from the initial need analysis along with the insight from the pharmacology department of the medical college, a design thinking workshop was organized involving key stakeholders. The team developed a prototype of the interactive hands-on workshop. The prototype was presented to CHOs, PHCOs, pharmacists, staff nurses, and medical officers managing the PHCs, and their feedback was elicited to finalize the workshop content and delivery.

### Workshop content

**Table 1:** Structure of workshop on medications for chronic disease

	Anti-hypertensive Medications	Diabetes Medications	Proton-pump inhibitors (PPI), Non-steroidal anti-inflammatory drugs (NSAID), Anti-histamines	Statins, Anti-anginal Medications, Anticoagulants, Asthma Medications
Background Information on Pathophysiology of Disease using Models	• Narrow vs. wide elastic pipe to mimic vasoconstriction and vasodilation of blood vessels • Squeezing water balloon to mimic force of heart pumping	• Pebbles (glucose) in a bag (bloodstream) • Small cup (insulin) to scoop pebbles (glucose) and place them into a can (body’s cells)	• Carbonated drink vs. water to compare stomach without PPI use and with PPI use	• Elastic tube with industry grease smeared in inner wall to resemble atherosclerosis. • Colored thermocol pieces (platelets) in sagittal cut-section of elastic tube • Ball in elastic tube to mimic blood clot • Narrowing of elastic pipe to resemble bronchoconstriction in asthma attack.
Overview of Medication Use, Mechanism of Action, Appropriate Dosage, and Side Effects	• Angiotensin converting enzyme (ACE) inhibitors • Angiotensin receptor blockers (ARBs) • Beta blockers • Thiazide diuretics • Calcium channel blockers	• Insulin • Biguanide - Metformin • Sulfonylureas - Glimepiride and Glibenclamide	• Pantoprazole • Paracetamol, • Ibuprofen, Diclofenac, Aceclofenac, Mefenamic acid • Cetirizine, Fexofenadine	• Atorvastatin and Rosuvastatin • Nitroglycerin • Aspirin • Albuterol and Theophylline
Role Play Scenarios	Audience members to guess the medication associated with side effect portrayed by facilitators (e.g. ACE inhibitor causing dry cough, thiazide diuretic causing increased urinary frequency)	Facilitator #1 to act out episode of hypoglycemia and facilitator #2 to display appropriate response using ‘15/15 rule’	Audience members to guess the medication associated with side effect portrayed by facilitators (e.g. antihistamine causing drowsiness, NSAID use causing gastric discomfort)	Facilitator #1 to act out episode of asthma attack and to display appropriate usage of albuterol inhaler, MDI and spacer
Clinical Vignettes	Audience members to guess appropriate anti-hypertensive and associated side effects based on patient description	Audience members to call out symptoms of diabetes in vignette and share appropriate anti-hyperglycemic and associated side effects based on patient description	Audience members to guess appropriate medication and associated side effects based on patient description	Audience members to guess appropriate medication and associated side effects based on patient description

The content of the training manual was divided into four topics: (1) anti-hypertensive medications, (2) medications for diabetes management, (3) proton-pump inhibitors, non-steroidal anti-inflammatory drugs, antihistamines, (4) statins, anti-anginal drugs, anticoagulants, and asthma medications. The workshop session plan corresponded to the four topics. Each topic was introduced with pathophysiology leading to the development of the specified condition illustrated using models and props (rubber water hose as blood vessels, water balloon as the heart, cans as cells, etc.). The workshop facilitator then explained the clinical use of medications to treat these conditions, the mechanism of action, appropriate dosage, potential side effects, and special considerations while using the medication (Table 1).

The training delivery was designed to fit into a participant-driven training model than an expert-led model. Though, the information was delivered through an oral demonstrative presentation in the local language (Kannada) with an associated PowerPoint presentation containing flow charts and diagrams to aid in participant understanding, at the same time, the workshop facilitators conducted various role play scenarios to depict either proper use of specific medications (proper use of asthma inhaler) and appropriate response to the adverse effect of medications (management of hypoglycemia in a diabetic patient). After the role-play scenarios, participants were asked quiz questions to assess their knowledge enhancement, if any. Each subtopic within the workshop ended with a discussion of case vignettes in which participants were asked to consider how they would respond to patients asking about medication use, response to side effects, and when referral to medical professionals is required.

### Evaluation

We conducted the workshop-based training program for the CHWs of two districts in rural Karnataka as an intervention with the pre- and post-intervention assessments. Participants completed a pre-intervention assessment immediately before the workshop, attended the workshop, and completed a post-intervention assessment immediately after the workshop (Supplemental file 2) (Supplemental file 3).

### Participant recruitment

We targeted a sample size of 131, assuming CHWs’ mean knowledge score would increase by 30% after the intervention at 95% confidence level and power of the study at 80% [16–18]. The district and sub-divisional health officers from the districts of Ramanagara and Madhugiri sent out invitations to all medical officers in both districts. A purposive census sampling approach was used to recruit all community health workers (CHWs) employed at 48 primary health centers (PHC) across the Ramanagara and Madhugiri districts. District health officers invited medical officers to nominate CHWs at their respective PHCs for participation in the workshop. The inclusion criteria required participants to be practicing community health workers (CHWs) with at least five months of work experience, aged 24 years or older, and have completed a minimum of 10th-grade education. There were no requirements regarding the length of time the CHW had resided in their designated community. The CHWs included in the training program and the study were CHOs, PHCOs, staff nurses at the primary care facilities, pharmacists and health inspectors. Upon the government district and sub-district health officers’ suggestions, participants from Ramanagara were invited to the central location of the health department’s district training center for the workshop. Government district and sub-district health officers in Madhugiri recommended that four workshops were conducted in four different PHCs for the CHWs from nearby health centers and villages to attend.

### Workshop implementation

The workshops were conducted in the year 2023 during the months of January and July as decided by the district health authorities. The workshop was three hours long. The organization that employs the research team bore the expenses for preparing the training module, A-V aids, and for travel of the facilitators. The training venue and refreshments were provided by the district health administration and health centers. The participants completed an informed consent prior to participation in the workshop, and participants were not provided any financial incentives to attend the training.

### Survey development and data collection

We intended to measure the change in knowledge, self-efficacy, and confidence among CHWs regarding common primary care medications. We developed a tool for knowledge assessment that included questions on indications for medication usage, common side effects, mechanism of action, dosage, and special considerations (Supplemental file 2) (Supplemental file 3). Questions related to prior sources of medication-related information and job responsibilities that require knowledge of medications were added to the questionnaire. Standard guidelines of MCQ preparation were followed according to andragogy methods [19]. Informed consent from all participants was collected in the pre-test online survey tool. The 31-question survey tool was composed of knowledge, self-efficacy, and confidence assessments (Table 2). The post-training assessment included the previously mentioned knowledge, self-efficacy and confidence-related questions as well as additional questions asking participant feedback on the quality, duration, and perceived usefulness of the half-day workshop based on the first two levels of Kirkpatrick model of training evaluation [20].

One hundred and forty participants completed the pre- and post-workshop assessment. The survey was self-administered under invigilation by the workshop facilitators.

**Table 2:** Components of pre-workshop and post-workshop assessment

Type of Assessment	Number of Questions	Cronbach’s alpha
Knowledge Assessment	20 questions	0.672
Self-Efficacy Assessment	5 questions	0.903
Confidence Assessment	6 questions	0.925

### Data analysis

Data analysis was conducted using SPSS® software version 20. Descriptive statistics for continuous variables, such as age, family income, and work experience, were reported as medians along with quartiles (Q1, Q3). Categorical variables like participants’ designation, highest education, number of household members, etc. were presented in percentages.

The knowledge portion of the assessment consisted of 20 questions requiring selection from multiple choice and matching a medication with its clinical use or side effect. Participants could receive a maximum of 20 points on the knowledge section. An adequate knowledge score was determined as ≥ 50% of questions correct.

Participants were asked to rank measures of self-efficacy in completing medication-related responsibilities as a CHW on a 5-point Likert scale in the pre-session and post-session assessment. The self-efficacy assessment was adapted from the validated General Self-Efficacy Scale [21]. High self-efficacy was determined to be an average score of 4 or greater on any 4 of the 5 questions. Measures of confidence in carrying out medication-related duties as a CHW were measured on a 5-point Likert scale in the pre-session and post-session assessment. High confidence was determined to be an average score of 4 or greater on any 4 of the 6 questions.

To assess changes in knowledge, self-efficacy, and confidence among participants regarding common primary care medication within the group, Wilcoxon’s signed rank test and McNemar test were employed. Pearson correlation test was employed to examine relationships between variables.

## Results

**Table 3:** Socio-demographic characteristics of the participants and occupational details

Characteristics	Median Value (Q1, Q3)
Age (years)	26 (24, 32)
Family Income (INR)	23,500 (12125, 40000)
Work experience (months)	9.5 (5, 28.5)
**Geographies of participating CHWs (Number of PHCs)**	**Number of CHWs (percentage of total participating CHWs)**
Madhugiri, Tumakuru (14 PHCs)	78 (55.7%)
Ramanagara (34 PHCs)	62 (44.3%)
**Designation of the participants**	**Number of CHWs (percentage of total participating CHWs)**
Community Health Officer (CHO)	103 (73.6%)
Primary Health Care Officer (PHCO)	29 (20.7%)
Health inspecting officer, Pharmacist	5 (3.6%)
Staff nurse	3 (2.1%)
**Highest Educational Level**	**Number of CHWs (percentage of total participating CHWs)**
10th standard	7 (5.0%)
12th standard	16 (11.4%)
Diploma	8 (5.7%)
Graduate - B.Sc. Nursing	102 (72.9%)
Graduate - other fields	3 (2.1%)
Masters - other fields	2 (1.4%)
Master of Science in Nursing	2 (1.4%)

CHW = community health worker, PHC = primary health center

Of 147 CHWs who attended the workshop, data from 140 CHWs (95.2% response rate) who submitted both the pre- and post-test assessments were included in the analysis. The median age of the participants was 26 years (24, 32) (1st quartile, 3rd quartile) and median duration of work experience as a CHW was 9.5 months (5, 28.5). Of the 140 participants, 78 (55.7%) worked in the Madhugiri taluk of Tumakuru district and 62 (44.3%) worked in the Ramanagara district. The majority of participating CHWs (102; 72.9%) had a Bachelor of Science in nursing degree–a prerequisite to be a CHO. Other degrees held by participating CHWs include completion of 10th grade (7; 5.0%), completion of 12th grade (16; 11.4%), a graduate degree in a field apart from nursing (3; 2.1%), diploma in a field apart from nursing (8; 5.7%), master’s degree in a field apart from nursing (2; 1.4%). All the PHCOs did not complete further education beyond 10th or 12th grade (Table 3).

**Table 4:** Distribution of prior knowledge, additional training, and work procedure

Characteristic	Frequency (%)
**Sources of information regarding medications**	
During Formal Education	119 (85.0%)
By interacting with other community health workers	54 (38.6%)
Through Field experience learning from community members	46 (32.9%)
Searching the internet or relevant books for the information	56 (40.0%)
**Job responsibilities related to medications**	
Obtaining/dispensing medications for patients	122 (87.1%)
Advising patients from their field practice areas on medications	137 (97.9%)
Encouraging patients on medication adherence	133 (95.0%)
Instructing patients with medication side effects to visit health facility	140 (100.0%)
**Did you receive any training related to any medications in the last one year?**	
Yes	46 (32.9%)
No	94 (67.1%)

The research team sought to understand prior sources of medication-related information for participating CHWs. CHWs could select all relevant sources in the pre-session survey. Less than half of the participating CHWs reported using information sources other than formal education. The majority of CHWs were unaware of or unable to access information post-education, indicating a need to connect CHWs with appropriate resources to further their knowledge in order to strengthen health literacy (Table 4).

CHWs reported having many significant job responsibilities that require knowledge of medications. Specifically, > 87% of CHWs are involved in obtaining and dispensing medications to patients, advising patients on medication, encouraging medication adherence, and referring patients with side effects to health facilities.

Regarding previous training related to any medications within the past year, only 46 (32.9%) reported having received some training related to any medications in the last year, largely as part of tuberculosis and maternal child health-related trainings.

**Table 5:** Changes in Knowledge, self-efficacy, confidence among participants regarding common primary care medications

Variable	Pre-Test Value	Post-Test Value	*p*-value
**Median (Q1,Q3) knowledge score (out of 20 points)**	11 (9,13)	16 (13,18)	< 0.01
Median (Q1,Q3) knowledge score on side effects of common medications (out of 7 points)	2 (1,3)	5 (3,6)	< 0.01
Median (Q1,Q3) knowledge score on clinical use of common medications (out of 8 points)	6 (4.25,7)	7 (6,8)	< 0.01
Median (Q1,Q3) knowledge score on contraindications, special considerations, appropriate dosing of common medications (out of 5 points)	3 (2,4)	4 (3,4)	< 0.01
**Number of participants (%) with adequate knowledge score regarding medications**	88 (62.8%)	129 (92.1%)	< 0.01
Number of participants (%) with adequate knowledge score regarding side effects of common medications	27 (19.3%)	97 (69.3%)	< 0.01
Number of participants (%) with adequate knowledge score regarding clinical use of common medications	114 (81.4%)	132 (94.3%)	< 0.01
Number of participants with adequate knowledge score regarding contraindications, special considerations, appropriate dosing of common medications	87 (62.1%)	121 (86.4%)	< 0.01
Participants with high self-efficacy regarding medications in their responsibilities as a community health worker	126 (90%)	140 (100%)	< 0.01
Participants with high confidence regarding medications in their responsibilities as a community health worker	127 (90.7%)	138 (98.5%)	< 0.01

The median knowledge score [IQR] on the pre-session assessment was 11 points (55%) [9, 13], while the median score on the post-session assessment was 16 points (80%) [13, 18], and this 25% increase in median knowledge score was statistically significant (*p* < 0.001). Further delineation of the knowledge score into knowledge of side effects of common medications revealed a pre-test median score of 2 points (maximum 7 points) [1, 3] that significantly increased to median score of 5 points [3, 6] on the post-session assessment (*p* < 0.001). Participants could receive a maximum of 8 points on questions related to clinical use of common medications.​​ There was a statistically significant increase in the median score from the pre-session assessment 6 [4.25, 7] to 7 on the post-session assessment [6, 8] (*p* < 0.002). CHWs could receive a maximum of 5 points on questions assessing knowledge of contraindications to prescribing medications to certain patient populations and appropriate medication dosage. The median score on the pre-session assessment was 3 [2, 4] and 4 [3, 4] on the post-session assessment (Table 5).

The percentage of participants with high self-efficacy increased by 10%, and the percentage of participants with high confidence increased by 8.5% after the workshop (Table 5). This emphasizes that CHWs should be provided with opportunities for continuing education to, not only increase their knowledge, but to also empower them in their professional roles.

**Table 6:** Participant reactions to the training program

Statement	Strongly Agree	Agree	Neither Agree nor Disagree	Disagree	Strongly Disagree
The training was easy to understand.	86 (61.4%)	50 (35.8%)	0	2 (1.4%)	2 (1.4%)
The training was engaging.	78 (55.8%)	52 (37.1%)	1 (0.7%)	6 (4.3%)	3 (2.1%)
The training was relevant to my responsibilities as a community health worker.	92 (65.8%)	44 (31.4%)	0	2 (1.4%)	2 (1.4%)
I am satisfied with the topics I have learned during the training program.	85 (60.7%)	50 (35.7%)	1 (0.7%)	2 (1.4%)	2 (1.4%)

The majority of participants strongly agreed with all four statements. Specifically, 97.2% of participants agreed that the training was easy to understand, 92.8% of participants felt the training was engaging, 97.2% of participants found the training relevant to CHW responsibilities, and 96.4% participants were satisfied with the topics they learned in the program (Table 6).

## Discussion

CHWs are the first point of contact between the community and the health care system. With the rise of non-communicable diseases (NCDs) in India, medication management has become one of the key responsibilities of CHWs. Previously, CHWs have been known to design educational materials for patients, advocate for them, and help patients feel comfortable reaching out to health providers [22, 23]. Prior trainings in India have aimed at equipping CHWs with the knowledge and skills to identify patient illnesses and encouraging patient participation [24–26]. CHWs have also assisted in screening for NCDs and building interpersonal connections with patients [27, 28]. As per current literature, this is the first CHW training in India centered around NCD medication uses, contraindications, and potential side effects.

Our study aimed to provide CHWs with the knowledge to assist patients with chronic diseases with medication-related concerns [27, 28]. The majority of participants were degree holders, primarily in nursing, and likely had prior exposure to pharmacology and medication-related content during their formal education. However, despite this background, our findings indicate that baseline knowledge scores were modest, and many participants reported limited practical exposure to medication management in the primary care or community setting. This workshop served as a continuous professional development (CPD) training aimed at contextualizing pharmacological knowledge for real-world community health work.

While CHWs cannot be expected to replace physicians, they can address day-to-day patient questions about when or how to take medications, monitor for unexpected side effects, and urgently refer patients to medical providers when necessary. Due to their sheer number and proximity to patients, CHWs interact with patients more frequently than physicians or nurses can. Other studies have also highlighted the shortage of providers who are knowledgeable on NCD medications in India [29]. After proper training, CHWs can aid patients in NCD medication management and increase patient health literacy related to medication use, thereby promoting greater medication adherence.

In our study, majority of participating CHWs reported that their routine tasks included dispensing medications to patients, responding to patients’ queries on medication uses and side effects, and referring patients to health facilities for medication-related side effects when needed. We also found that the level of CHWs’ self-reported involvement in tasks related to medications was significantly greater than what has been previously described in other reviews of CHW responsibilities (< 20% of 30 studies we reviewed, noted medication management as a CHW responsibility) [30]. However, fewer than one-third of participating CHWs in our study had received training on medication usage in the past year. These were reportedly limited to the programmatic training they received in the past year. For example, CHWs only learn about tuberculosis (TB) medications during TB-related training. The need for continued management of NCD medications has been cited in other studies aimed at NCD screening. In one study centered on CHW-led risk reduction, though all patients who screened positive for hypertension were started on anti-hypertensives, modification, and escalation of the medication regimen remained a significant limitation to blood pressure control. In response to this barrier, this study proposed training CHWs on anti-hypertensives so that they could adjust patient regimens as needed [31].

This synchronous half-day training on NCD medications was effective in significantly increasing participant knowledge. After attending the training, participants had a better understanding of medication uses, side effects, and contraindications as compared to before the training. Particularly, during the qualitative feedback at the end of sessions, participants appreciated the use of visual aids (e.g. models, diagrams) to illustrate the pathophysiology of chronic conditions such as diabetes and hypertension and the mechanism of action of medications used to treat these conditions. Due to the informal nature of prior CHW exposure to medication-related information (e.g. through field experience, and interactions with other CHWs), CHWs’ understanding of medication uses may have been purely associational. Through the mechanistic style of teaching in the workshop that involved breaking down complex subjects into smaller, more manageable components and understanding how those components interact and function together, CHWs could probably develop a foundational understanding of physiology and pharmacology. This would not only increase CHWs’ competence in triaging medication-related queries but would enable CHWs to better grasp future training on other topics in disease prevention and management. Further, the workshop model incorporated role-play scenarios, quiz questions, and case vignettes that all relied on audience participation. These are in line with adult learning techniques of gamification, active self-directed learning and placing relevance and immediate application into the specific learning outcomes. After receiving information on various medications, the CHWs had to synthesize this content and apply it to real-world scenarios. The patient cases included in the training were simplified and adapted based on feedback from medical officers who work in the community with CHWs. Thus, the patient scenarios mimicked the real patients that CHWs interact with daily. For this reason, the majority of CHWs noted that the workshop content was applicable to their duties and engaging throughout.

Our workshop design was similar to another CHW training on chronic obstructive pulmonary disease (COPD) management in that both used videos and the ‘teach-back method’, followed by assessment of CHWs’ knowledge [32]. However, our study also used physical models, included role play scenarios, and assessed CHW self-efficacy and confidence in addition to knowledge.

After completion of the training, workshop participants reported a significant increase in self-efficacy and confidence in completing medication-related tasks. Particularly, this workshop aimed to help CHWs feel confident delineating standard patient questions about medications from more complex patient presentations that require referral to a health center. With adequate training, CHWs can serve as a reliable bridge between patients and the healthcare system, ensuring that patients only visit a health center when their medication-related questions cannot be answered by a CHW. Further, CHWs can be instrumental in increasing medication adherence within the community. Due to frequent interactions, community members are often more comfortable speaking with a CHW than a formal healthcare provider. Thus, as CHWs become more confident in overseeing medication regimens, they can encourage greater medication adherence within the community.

Although this training was effective in its goal of increasing CHW knowledge of medications, since the workshop was synchronous, CHWs had to leave their busy work schedules for an entire day to attend the workshop in-person and allot commute time. In order to more easily incorporate this training into CHW’s strenuous work schedules, the workshop could be conducted within PHCs rather than at a central location that requires CHWs to travel. To further reduce human resources required and encourage sustainability of the training program, CHWs who attend the workshop could conduct the workshop for their peers in the future. This would allow for CHWs to augment their leadership and public speaking skills. Additionally, local workshop facilitators could more easily develop patient case vignettes based on patient complaints and commonly used medications in that particular community.

## Study limitations

We did not include a control arm in our study because our objective was not to test attribution of the results to the training program, but to assess improvement of knowledge among the participants, if at all. However, due to the absence of a similar training program, it can be assumed that the change in medication related knowledge in the absence of this training would be negligible. Further, the measurement of self-efficacy was based on an adaptation of the General Self-Efficacy Scale (GSES), which captures general perceptions of one’s ability to cope with challenges rather than role-specific competencies related to community health work [21]. While the GSES is a validated and reliable instrument, it may not fully reflect the nuanced aspects of CHW self-efficacy—such as confidence in performing medication-related tasks or counseling patients on chronic disease management. Future research should consider employing or developing a CHW-specific self-efficacy scale that better captures behavioral and context-specific dimensions of confidence. Next, participants were assessed on their knowledge of medications immediately before and immediately after the training. Thus, our study did not assess knowledge retention in the weeks and months following the training. Thus, to determine whether this workshop format allows for long-term retention and application of the knowledge, CHW knowledge of medications should be assessed at interval time points (1 month, 3 months) after completion of the training. 

## Future directions

Future iterations of this training should allow for increased scalability, retention of knowledge, and reinforcement. Considering CHWs are frequently confronted with medication-related tasks, a periodic refresher training for all CHWs and healthcare providers on medication usage, dosages, side effects, and contraindications can be developed and implemented in partnership with the state and national institute for public health training. Furthermore, considering the diversity in educational backgrounds and generally unsatisfactory levels of health literacy among CHWs, medication-related training must be standardized to ensure CHWs are adequately prepared to respond to the healthcare needs of the community. To complement the workshop-based training, an asynchronous program through a mobile-based app platform can be developed for training reinforcement and assessment. This way, CHWs could access the training at their fingertips on their own time and refer back to the training when questions arise in patient care. In the digital era, CHWs should be equipped with the skills and resources to collect and monitor patient data using technological devices and online platforms. Expanding the CHW skill set to include technology use can also increase community trust and uptake of services [33]. Considering low baseline digital literacy levels in CHWs, digital training could increase CHW knowledge while simultaneously increasing CHW comfort in using technology for other patient care responsibilities.

The intended long-term outcome of medication training for CHWs is increased medication adherence and prevention of complications from chronic diseases due to poor medication adherence and medication-related side effects. As this workshop is delivered to a greater number of CHWs, CHWs should be observed for how they utilize the information, counsel the patients in the communities they serve and prevent complications. Patients in the community can be assessed for any change in the practices of medication adherence. Thus, the assessment of the training could be expanded to level 3 (behavior) and level 4 (intended results) of the Kirkpatrick model [20]. A review conducted on CHW training in low and middle-income countries similarly found that most studies evaluated knowledge outcomes rather than the impact on community health as a result of the training [34].

## Conclusion

This study demonstrates that a focused training workshop on chronic disease medications was effective in increasing the knowledge, self-efficacy, and confidence of community health workers (CHWs) in two districts of rural Karnataka. By strengthening CHWs’ understanding of medication use, side effects, and contraindications, the workshop equipped them to better triage medication-related concerns and support patients in managing chronic conditions. These findings emphasize the pivotal role of CHWs as the first point of contact in India’s healthcare system—particularly as the country faces a rapid shift from infectious to noncommunicable diseases.

For policymakers and program implementers, this study provides a scalable and sustainable model for capacity building that can be integrated into existing CHW training programs across India. Expanding this model nationally could strengthen medication management at the community level, improve treatment adherence, and alleviate the burden on higher-level health facilities. Moreover, similar training programs could be adapted for other low- and middle-income countries seeking to empower frontline health workers in chronic disease care.

In the future, we propose distribution of the training through a mobile app platform and assessment of how well CHWs retain the knowledge, whether CHW interactions with community members are impacted as a result of the training, and corresponding changes in the level of medication adherence in the community.

## Supplementary Information

Below is the link to the electronic supplementary material.

Supplementary Material 1

Supplementary Material 2

Supplementary Material 3

## Data Availability

The datasets used and analyzed during the current study are available from the corresponding author on reasonable request.

## Abbreviations

ACE: Angiotensin-Converting Enzyme
ARB: Angiotensin Receptor Blocker
CHC: Community Health Center
CHW: Community Health Workers
COPD: Chronic Obstructive Pulmonary Disease
CPHC: Comprehensive Primary Health Care
HWC: Health and Wellness Center
MCQ: Multiple-Choice Question
NCD: Non-Communicable Diseases
NFHS: National Family Health Survey
NSAID: Non-steroidal anti-inflammatory drug
PHC: Primary Health Center
PHCO: Primary Health Care Officers
PPI: Proton-Pump Inhibitor
TB: Tuberculosis
