# Supplementary material for: Evaluation of a synchronous training program on common primary care medications for community health workers in Karnataka, India
Source: BMC Health Serv Res. 2026 Feb 4;26:250. doi: 10.1186/s12913-025-13754-x (PMC12905942; doi:10.1186/s12913-025-13754-x)
Supplement: Supplementary file 1 — Supplementary Material 1 [file 12913_2025_13754_MOESM1_ESM.docx]

**Interview Guide for Preliminary Needs Assessment**

Greet the participant, introduce yourself and explain again the following:

Objective of the interview: To list the medications available at their primary care facility and to enquire about any training they received regarding medications.

Expected interview/discussion time: Approximately 25-30 minutes

With informed consent, interviewer turns on the digital recorder and starts the conversation

**Probes:**

1. Ask their name, age, education, years of experience, their place of work, etc.
2. Probe on the activities they do as a health care worker/provider.
3. Request them to elicit their responsibilities involving medications both at the facility and in the communities they serve.
4. Enquire about any specific training they have received regarding primary care medications.

Request them to show all the medications that are present at the primary healthcare centre (PHC) or the Health and Wellness Centre (HWC).

Click photos of each medication (front and back).

Make a list of medications available and the Essential Drug List (EDL) board, if available, at the PHCs.

Thank the participant, exchange contact information and end the interaction.
