# Supplementary material for: Evaluation of a synchronous training program on common primary care medications for community health workers in Karnataka, India
Source: BMC Health Serv Res. 2026 Feb 4;26:250. doi: 10.1186/s12913-025-13754-x (PMC12905942; doi:10.1186/s12913-025-13754-x)
Supplement: Supplementary file 2 — Supplementary Material 2 [file 12913_2025_13754_MOESM2_ESM.pdf]

English Version - Medication Pre-Training Assessment Tool

* Indicates required question

Consent Form

Study title: Ramaiah Chronic Disease Medication Training

Principal investigator: Dr. Ananth Ram

Co-Investigator: Ashwini Deshpande

Contact number: 080-23605190 Extn: 268
Dear participants,

As you are a Frontline Health Worker (CHO or PHCO) working closely with patients with Hypertension (BP) or Diabe (high blood sugar), you are invited to be a part of this chronic disease medication training. The project is being conducted by the Ramaiah International Center for Public Health Innovations (RICPHI): a unit of Gokula Shiksha Pratishthan (Medical), Bangalore.

Your participation in this survey is completely voluntary and there is no cost to participate in the study.

Objective of the Study: This study aims to provide community health workers with information on commonly used drugs for chronic diseases (diabetes, hypertension, asthma, high cholesterol). The study will assess how effective this training is in improving community health workers' medication knowledge, confidence and self-efficacy.

Involvement: You will be asked to complete an online module (via SHARPEN Platform) with videos and a series of MCQs on drugs. Before and after the training, you will be asked questions about your confidence in your responsibilities, strength of relationship with patients and knowledge about medicines.

Benefits: Online medication training gives you information about the clinical use, appropriate dosage and side effec of chronic disease medications. You will be better equipped to advise patients in the community on appropriate medication use, identify side effects from medications, refer patients to the PHC Medical Officer when necessary a encourage medication adherence among patients.

By signing this form, you consent to the use and sharing of the following information: your contact information, personal demographic information (age, occupational status) and questionnaire responses.

Please fill in your name and check the box below.

1. Name *

2. Today's Date *

Example: January 7, 2019

3. Phone No. *

4. I have read the above form and agree to participate in the ACE-ME medication training program. *

Mark only one oval.

- Yes
- No

5. 1. Age *

6. 2. Which district do you work in? *

Mark only one oval.

- Tumakuru
- Ramanagara

7. 3. Which primary health center (PHC) do you work in? *

8. 4. What is your occupation? *

Mark only one oval.

- ( ) CHO
- ( ) PHCO
- ( ) Staff Nurse
- ( ) Pharmacist
- ( ) Other
- ( ) Other: ______________________

9. 5. How many years have you served in your current occupation? *

____

10. 6. What is the highest degree of education you have attained? *

____

11. 7. How many children do you have? *

Mark only one oval.

- ( ) 0
- ( ) 1
- ( ) 2
- ( ) 3
- ( ) 4
- ( ) 5
- ( ) 6+

12. 8. How many members are there in your household? *

Mark only one oval.

- 1
- 2
- 3
- 4
- 5
- 6
- 7
- 8+

13. 9. What is your family's monthly income? *

____

14. 10. Where do you get information about commonly available medications? (select all that apply) *

Check all that apply.

- [ ] Never learned about medications
- [ ] During education
- [ ] By talking to other community health workers (CHO, ASHA, PHCO)
- [ ] Through experience (talking with patients in the community)
- [ ] By searching the internet or related books for information

15. 11. In the last one year, have you received any training on medicines? *

Mark only one oval.

- Yes
- No

Knowledge

16. 1. Please select the relevant side-effect of the following medicines:
(Please scroll right to see all answer options)

Mark only one oval per row.

	Dry cough	Swelling of feet	Low blood sugar	Decreased appetite	Low potassium: Fatigue, cramps and fast heart rate	Drowsiness	I'm no sure
**Amlodipine (calcium channel blocker)**	⬭	⬭	⬭	⬭	⬭	⬭	⬭
**Enalapril (ACE inhibitor)**	⬭	⬭	⬭	⬭	⬭	⬭	⬭
**Hydrochlorothiazide (diuretic)**	⬭	⬭	⬭	⬭	⬭	⬭	⬭
**Insulin**	⬭	⬭	⬭	⬭	⬭	⬭	⬭
**Metformin**	⬭	⬭	⬭	⬭	⬭	⬭	⬭
**Cetirizine**	⬭	⬭	⬭	⬭	⬭	⬭	⬭

17. 2. Please match the following drugs with their clinical use.
(Please scroll right to see all answer options)

Mark only one oval per row.

	Asthma attack	Diabetes	High cholesterol	High blood pressure	Gastritis or gastroesophageal reflux disease (GERD)	Angina (chest pain)	Allergies	[illegible]
**Nitroglycerin**	○	○	○	○	○	○	○	
**Atenolol (beta blocker)**	○	○	○	○	○	○	○	
**Pantoprazole (proton pump inhibitor)**	○	○	○	○	○	○	○	
**Salbutamol**	○	○	○	○	○	○	○	
**Glimepiride**	○	○	○	○	○	○	○	
**Folate (Vitamin B9)**	○	○	○	○	○	○	○	
**Atorvastatin (statin)**	○	○	○	○	○	○	○	

18. 3. Why should patients take aspirin regularly?

Mark only one oval.

- A. Aspirin prevents the formation of harmful blood clots. It reduces the chances of heart attack or stroke.
- B. Aspirin dilates blood vessels and reduces stress on the heart during episodes of chest pain.
- C. Aspirin slows the heart rate and allows the heart to pump more blood each time. Thus, all parts of the body get adequate supply of blood and oxygen.
- D. Aspirin helps the body get rid of excess sodium and water that can cause episodes of chest pain.
- E. I'm not sure

19. 4. Which patients are not allowed to take beta blockers for hypertension treatment?

Mark only one oval.

- A. Patients with heart disease
- B. Asthma patients
- C. Patients over 60 years of age
- D. Patients with renal disease
- E. I'm not sure

20. 5) How can a patient immediately resolve symptoms of low blood sugar (hypoglycemia)?

Mark only one oval.

- A. Rock sugar, candy, or sugar
- B. Eat more fiber
- C. Stop taking Glucomet [metformin]
- D. Stop taking Amaryl [glimepiride]
- E. I'm not sure

21. 6) Which of the following drugs increases the amount of insulin released by the pancreas and can be taken by patients along with Glucomet [Metformin]?

Mark only one oval.

- A. Insulin
- B. Glucan D Powder
- C. Glimepiride
- D. Pregeb [Pregabalin]
- E. I'm not sure

22. 7) Which of the following medicines helps stop headache and reduce fever?

Mark only one oval.

- A. Vitamin D
- B. Glucomet (Metformin)
- C. Nitroglycerin
- D. Paracetamol
- E. I'm not sure

23. 8. What are the side effects of ibuprofen after continuous and long term use?

Mark only one oval.

- A. Blurry vision
- B. Bleeding in stomach
- C. Loss of balance
- D. Weight loss

24. 9. A patient consumes Asthalin (Salbutamol) every hour. What do you expect to happen?

Mark only one oval.

- A. There is no change because patients usually take Asthalin every hour.
- B. Patients experience symptoms of an asthma attack.
- C. Patients experience symptoms of Asthalin overdose (rapid heartbeat, tremors) because Astaline should not be inhaled more than three times a day.
- D. Asthalin should be taken as a tablet rather than by inhaler.
- E. I'm not sure

1. In which of the following areas would you like additional training?

25. 1a. Understanding how medicines work *

Mark only one oval.

- Yes
- No

26. 1b. Determining the medical use of drugs *

Mark only one oval.

- Yes
- No

27. 1c. Identifying unexpected drug side effects in patients *

Mark only one oval.

- Yes
- No

28. 1d. Understanding which patients should not take certain medications *

Mark only one oval.

- Yes
- No

29. 1e. Encouraging medication adherence among community members *

Mark only one oval.

- Yes
- No

2) Which medication-related activities are you involved in?

30. 2a. Do you deliver medicines to patients? *

Mark only one oval.

- Yes
- No

31. 2b. Do you counsel your patients on medications? *

Mark only one oval.

- Yes
- No

32. 2c. Do you encourage medication adherence in your patients? *

Mark only one oval.

- Yes
- No

33. 2d. Have you suggested that patients experiencing unexpected side effects from medications visit a doctor?

Mark only one oval.

- Yes
- No

Self-Efficacy

[Rate on a scale of 1-5 (1 = Strongly Disagree, 2 = Disagree, 3 = Don't Know, 4 = Agree, 5 = Strongly Agree)]

Please scroll right to see all answer options.

34. 1. Describe your self-efficacy as a community health worker. *
Please scroll right to see all answer options.

Mark only one oval per row.

	1 = Strongly disagree	2 = Disagree	3 = I'm unsure	4 = Agree	5 = Strongly agree
**I am confident that I can respond competently to unexpected health events in patients.**	◯	◯	◯	◯	◯
**I believe that I can solve most problems at work.**	◯	◯	◯	◯	◯
**If I don't immediately have an answer to a patient's question, I trust that I can find the right information.**	◯	◯	◯	◯	◯
**I can solve problems calmly because of my past training and experience.**	◯	◯	◯	◯	◯
**I feel confident advising community members on why to use medications, how to use them, and possible side effects of using them.**	◯	◯	◯	◯	◯

Confidence Regarding Discussing Medications

[Rate on a scale of 1-5 (1 = Strongly Disagree, 2 = Disagree, 3 = Don't Know, 4 = Agree, 5 = Strongly Agree)]

Please scroll right to see all answer options.

35. 2. Describe your confidence in discussing medications. *
Please scroll right to see all answer options.

Mark only one oval per row.

	1 = Strongly disagree	2 = Disagree	3 = I'm unsure	4 = Agree	5 = Strongly agree
**I believe I am able to effectively discuss health information with patients.**					
**I believe I can effectively answer questions about medications with anyone in the community who has any health condition.**					
**I believe community members trust the information I give them.**					
**I am seen as knowledgeable by community members.**					
**I believe that when they have any health problem, community members should ask me about it first.**					
**I care deeply about improving the health of those with health conditions (diabetes, hypertension) in the community.**					

This content is neither created nor endorsed by Google.

Google Forms
