# Supplementary material for: Evaluation of a synchronous training program on common primary care medications for community health workers in Karnataka, India
Source: BMC Health Serv Res. 2026 Feb 4;26:250. doi: 10.1186/s12913-025-13754-x (PMC12905942; doi:10.1186/s12913-025-13754-x)
Supplement: Supplementary file 3 — Supplementary Material 3 [file 12913_2025_13754_MOESM3_ESM.pdf]

Medication Post-Training Assessment Tool - Ramanagara

* Indicates required question

1. Name *

2. Phone No. *

3. Which Primary Health Center (PHC) do you work in? *

Perceived Knowledge Changes

[Rate on a scale of 1-5 (1 = Strongly Disagree, 2 = Disagree, 3 = Don't Know, 4 = Agree, 5 = Strongly Agree)]

4. 1. Describe the changes in your knowledge after the training.
Please scroll right to see all answer options.

Mark only one oval per row.

	1 = Strongly disagree	2 = Disagree	3 = I don't know	4 = Agree	5 = Strongly agree
**I have a stronger understanding of the cause of hypertension and anti-hypertensive medications than I had before the training.**					
**I have a stronger understanding of the causes of diabetes and how diabetes medications work than I did prior to the training.**					
**I have a stronger understanding of how statins, nitroglycerin, aspirin, asthma medications work than I did before training.**					
**I have a stronger understanding of how generic drugs (ibuprofen, paracetamol, antihistamines, etc.) work than I had before the training.**					
**I am much more prepared to advise my patients on how and when to use medications compared to before receiving the training.**					
**I am more prepared to recognize adverse side effects from medications than I was before training.**					

Knowledge

5. 1. Please select the relevant side-effect of the following medicines:
(Please scroll right to see all answer options)

Mark only one oval per row.

	Dry cough	Swelling of feet	Low blood sugar	Decreased appetite	Low potassium: fatigue, cramps and fast heart rate	Drowsiness	I'm no sure
**Amlodipine (calcium channel blocker)**	⬭	⬭	⬭	⬭	⬭	⬭	⬭
**Enalapril (ACE inhibitor)**	⬭	⬭	⬭	⬭	⬭	⬭	⬭
**Hydrochlorothiazide (diuretic)**	⬭	⬭	⬭	⬭	⬭	⬭	⬭
**Insulin**	⬭	⬭	⬭	⬭	⬭	⬭	⬭
**Metformin**	⬭	⬭	⬭	⬭	⬭	⬭	⬭
**Cetirizine**	⬭	⬭	⬭	⬭	⬭	⬭	⬭

6. 2. Please match the following drugs with their clinical use. (Please scroll right to see all answer options)

Mark only one oval per row.

	Asthma attack	Diabetes	High cholesterol	High blood pressure	Gastritis or gastroesophageal reflux disease (GERD)	Angina (chest pain)	Allergies	[illegible]
**Nitroglycerin**								
**Atenolol (beta blocker)**								
**Pantoprazole (proton pump inhibitor)**								
**Salbutamol**								
**Glimepiride**								
**Folate (Vitamin B9)**								
**Atorvastatin (statin)**								

7. 3. Why should patients take aspirin regularly?

Mark only one oval.

- A. Aspirin prevents the formation of harmful blood clots. It reduces the chances of heart attack or stroke.
- B. Aspirin dilates blood vessels and reduces stress on the heart during episodes of chest pain.
- C. Aspirin slows the heart rate and allows the heart to pump more blood each time. Thus, all parts of the body get adequate supply of blood and oxygen.
- D. Aspirin helps the body get rid of excess sodium and water that can cause episodes of chest pain.
- E. I'm not sure

8. 4. Which patients are not allowed to take beta blockers for hypertension treatment?

Mark only one oval.

- A. Patients with heart disease
- B. Asthma patients
- C. Patients over 60 years of age
- D. Patients with renal disease
- E. I'm not sure

9. 5) How can a patient immediately resolve symptoms of low blood sugar (hypoglycemia)?

Mark only one oval.

- A. Rock sugar, candy, or sugar
- B. Eat more fiber
- C. Stop taking Glucomet [metformin]
- D. Stop taking Amaryl [glimepiride]
- E. I'm not sure

10. 6) Which of the following drugs increases the amount of insulin released by the pancreas and can be taken by patients along with Glucomet [Metformin]?

Mark only one oval.

- A. Insulin
- B. Glucan D Powder
- C. Glimepiride
- D. Pregeb [Pregabalin]
- E. I'm not sure

11. 7) Which of the following medicines helps stop headache and reduce fever?

Mark only one oval.

- A. Vitamin D
- B. Glucomet (Metformin)
- C. Nitroglycerin
- D. Paracetamol
- E. I'm not sure

12. 8. What are the side effects of ibuprofen after continuous and long term use?

Mark only one oval.

- A. Blurry vision
- B. Bleeding in stomach
- C. Loss of balance
- D. Weight loss

13. 9. A patient consumes Asthalin (Salbutamol) every hour. What do you expect to happen?

Mark only one oval.

- A. There is no change because patients usually take Asthalin every hour.
- B. Patients experience symptoms of an asthma attack.
- C. Patients experience symptoms of Asthalin overdose (rapid heartbeat, tremors) because Astaline should not be inhaled more than three times a day.
- D. Asthalin should be taken as a tablet rather than by inhaler.
- E. I'm not sure

Self-Efficacy

[Rate on a scale of 1-5 (1 = Strongly Disagree, 2 = Disagree, 3 = Don't Know, 4 = Agree, 5 = Strongly Agree)]

Please scroll right to see all answer options.

14. 1. Describe your self-efficacy as a community health worker. *
Please scroll right to see all answer options.

Mark only one oval per row.

	1 = Strongly Disagree	2 = Disagree	3 = I'm unsure	4 = Agree	5 = Strongly Agree
**I am confident that I can respond competently to unexpected health events in patients.**	◯	◯	◯	◯	◯
**I believe that I can solve most problems at work.**	◯	◯	◯	◯	◯
**If I don't immediately have an answer to a patient's question, I trust that I can find the right information.**	◯	◯	◯	◯	◯
**I can solve problems calmly because of my past training and experience.**	◯	◯	◯	◯	◯
**I feel confident advising community members on why to use medications, how to use them, and possible side effects of using them.**	◯	◯	◯	◯	◯

Confidence Regarding Discussing Medications

[Rate on a scale of 1-5 (1 = Strongly Disagree, 2 = Disagree, 3 = Don't Know, 4 = Agree, 5 = Strongly Agree)]

Please scroll right to see all answer options.

15. 2. Describe your confidence in discussing medications. *
Please scroll right to see all answer options.

Mark only one oval per row.

	1 = Strongly disagree	2 = Disagree	3 = I'm unsure	4 = Agree	5 = Strongly agree
**I believe I am able to effectively discuss health information with patients.**	◯	◯	◯	◯	◯
**I believe I can effectively answer questions about medications with anyone in the community who has any health condition.**	◯	◯	◯	◯	◯
**I believe community members trust the information I give them.**	◯	◯	◯	◯	◯
**I am seen as knowledgeable by community members.**	◯	◯	◯	◯	◯
**I believe that when they have any health problem, community members should ask me about it first.**	◯	◯	◯	◯	◯
**I care deeply about improving the health of those with health conditions (diabetes, hypertension) in the community.**	◯	◯	◯	◯	◯

Please press "Submit"

16. Do not type anything here. Please press "Submit" below. *

____

Do not edit this field, click on SUBMIT

The field below is needed by Form Timer and must NOT be modified. If you modify this key your answer will not be recorded.

17. Fingerprint - DO NOT EDIT *

This content is neither created nor endorsed by Google.

Google Forms
